# Plasticity of the bony carotid canal and its clinical use for assessing negative remodeling of the internal carotid artery

**DOI:** 10.1371/journal.pone.0261235

**Published:** 2021-12-15

**Authors:** Yuki Oichi, Yohei Mineharu, Yuji Agawa, Takaaki Morimoto, Takeshi Funaki, Yasutaka Fushimi, Kazumichi Yoshida, Hiroharu Kataoka, Susumu Miyamoto

**Affiliations:** 1 Department of Neurosurgery, Kyoto University Graduate School of Medicine, Kyoto, Japan; 2 Department of Neurosurgery, Kokura Memorial Hospital, Kokura, Japan; 3 Department of Neurosurgery, Amagasaki General Medical Center, Amagasaki, Japan; 4 Department of Diagnostic Imaging and Nuclear Medicine, Kyoto University Graduate School of Medicine, Kyoto, Japan; 5 Department of Neurosurgery, National Cerebral and Cardiovascular Center, Suita, Japan; Kaohsuing Medical University Hospital, TAIWAN

## Abstract

**Background and objective:**

It has long been believed that the bony carotid canal has no plasticity and that a small canal represents a hypoplastic internal carotid artery. We aimed to show whether the carotid canal can narrow according to morphological changes in the internal carotid artery.

**Materials and methods:**

The carotid canal diameter was longitudinally measured in seven individuals who underwent carotid artery ligation. As moyamoya disease is known to be associated with negative remodeling of the internal carotid artery, the carotid canal diameter was measured in 106 patients with moyamoya disease, and an association with the outer diameter of the internal carotid artery or a correlation with the disease stage was investigated. The carotid canal was measured by computed tomography (106 patients), and the outer diameter of the artery was measured by high-resolution magnetic resonance imaging (63 patients). The carotid canal area was calculated by the product of the maximum axial diameter and its perpendicular diameter.

**Results:**

All seven patients who underwent carotid artery ligation showed narrowing of the carotid canal, and the carotid canal area decreased by 12.2%–28.9% during a mean follow-up period of 4.2 years. In patients with moyamoya disease, the carotid canal area showed a linear correlation with the outer area of the internal carotid artery (r = 0.657, p < 0.001), and a negative correlation with the disease stage (*ρ* = −0.283, p < 0.001).

**Conclusion:**

The bony carotid canal has plasticity, and its area reflects the outer area of the internal carotid artery, therefore, it can be used to assess the remodeling of the carotid artery. A narrow carotid canal may not necessarily indicate hypoplastic internal carotid artery.

## Introduction

The bony carotid canal is a passage within the petrous temporal bone that transmits the internal carotid artery (ICA) and sympathetic plexus. Its inferior opening is called the carotid foramen, which is situated anterior to the jugular fossa and medial to the carotid plate. This canal has been recognized as a stable structure that does not change shape; thus, hypoplasia of the bony carotid canal has been used to determine hypoplasia of the ICA [1, 2]. However, no study has tested the possibility that the bony carotid canal may change shape according to ICA size.

To determine whether the bony carotid canal has plasticity, we measured temporal changes in carotid canal diameter in patients who underwent common carotid artery ligation, which causes atrophy of the ICA. Additionally, to determine whether carotid canal remodeling occurs even if morphological changes in the ICA are subtle and slow, we selected patients with moyamoya disease (MMD) as a study population. MMD is an idiopathic arteriopathy in which the terminal portion of the ICA suffers progressive stenosis, and the reduced blood flow induced by the stenosis is compensated by fine collaterals called moyamoya vessels [3]. Recent advances in magnetic resonance imaging (MRI) enabled us to characterize morphological changes in the intracranial arteries. It has been well recognized that MMD is characterized by a small outer diameter of the ICA and concentric stenosis, whereas atherosclerosis is characterized by ICA enlargement and eccentric stenosis [4]. Furthermore, Kuroda et al. showed that the outer diameter of the ICA decreases according to advancement in Suzuki’s disease stage [5], suggesting negative (inward) ICA remodeling.

Thus, in the present study, we aimed to show that the bony carotid canal can remodel according to morphological changes in the ICA, using two different study cohorts: individuals who underwent carotid artery ligation and patients with MMD.

## Study population and methods

### Study population

This study was approved by the Ethics Committee of the Institutional Review Board of Kyoto University (approval numbers: G1109, G0342, and R2088). We retrospectively reviewed seven patients who underwent carotid artery ligation between January 2007 and August 2014 in Kyoto University Hospital. These patients underwent operation to treat large intracranial aneurysms. MMD and unilateral MMD were diagnosed according to the diagnostic criteria of the Research Committee on Moyamoya Disease and were generally evaluated using digital subtraction angiography [6, 7]. We included 106 patients with MMD (23 were unilateral cases) who underwent radiological evaluation between July 2011 and August 2020. Individuals under the age of 17 years were considered children. Written informed consent was obtained from all patients and legal guardians.

### Radiological evaluations

To evaluate the area of the bony carotid canal, three dimensional images of thin slice computed tomography (CT) were reconstructed on a workstation (AquariusNetStation Terarecon Inc., San Mateo, CA, USA). The carotid canal of the horizontal part was sectioned perpendicularly to its long axis, and the maximum diameter and its perpendicular diameter were measured in 212 hemispheres of 106 patients with MMD (S1 Fig). Using these diameters, the carotid canal area was calculated by applying a modified formula for the area of an ellipse, where area = (maximum diameter) x (perpendicular diameter) x pi/4. In previous papers, the carotid canal diameter was measured along the horizontal axis of it [8], but we have adopted this method because the carotid canal is shaped like an ellipse and the deformation could be different in the major and minor axes. Rather than measuring only one cross-section, the horizontal axis, it was considered more accurate to measure two orthogonal cross-sections. The inter-rater reliability between the two examiners for this measurement method was analyzed using the intraclass correlation coefficient. The maximum outer diameter and its perpendicular outer diameter of the ICA were also measured in a similar way from high-resolution black-blood MRI images using the workstation (AquariusNetStation) for 126 hemispheres in 63 individuals (56 bilateral and 7 unilateral cases). We adopted the same method of workstation-based measurement of the ICA because we considered that the tilt (angle) of the carotid canal and that of the ICA are different and therefore the comparison of the horizontal axial section of each structure cannot be so accurate. These diameters of the ICA were measured just before it branched to the anterior cerebral artery and the middle cerebral artery. We did not use the outer diameter of the ICA at the level of the bony carotid canal (horizontal part, C4) because of inaccurate measurement that was likely related to bone artifact. To assess the plasticity of the bony carotid canal, serial measurements of the carotid canal were available for 7 individuals who underwent carotid artery ligation and 4 patients with unilateral MMD who developed contralateral progression. The acquisition parameters of CT and high-resolution black-blood MRI are shown in S1 Table.

### Statistical analyses

Statistical analyses were performed using GraphPad Prism software (version 8.2.1) or JMP Pro statistical software (version 14.0.0; SAS Institute Inc., Cary, NC). Clinical characteristics were compared between cases and controls using Student’s *t-*test for continuous variables and Fisher’s exact test for categorical variables. Pearson’s correlation coefficient (r) was used to assess the linear correlation between continuous variables, after checking the normality of each variable. The normality was checked by the normal quantile-quantile (Q-Q) plot and Shapiro-Wilk test. Spearman’s correlation coefficient (*ρ*) was used when an ordinal variable is included. A p-value of < 0.05 was considered statistically significant. The intraclass correlation coefficient was calculated using the Evaluating the Measurement Process method of the JMP Pro statistical software.

## Results

### Negative remodeling of the bony carotid canal after carotid artery ligation

The characteristics of the study population are shown in Table 1. The inter-rater reliability between the two independent examiners for the measurement of the bony internal carotid canal and the ICA was assessed in 40 hemispheres of the patients with MMD. The intraclass correlation coefficients of the maximum diameter, its perpendicular diameter, and area of the bony carotid canal were 0.973, 0.971 and 0.986, respectively. Those of the ICA were 0.838, 0.828 and 0.805, respectively. All coefficients were > 0.8, which is considered highly reliable. The mean (standard deviation) of difference in measurement between the two examiners for the carotid canal diameter and the outer diameter of the ICA was 0.112 (0.088) mm and 0.360 (0.290) mm respectively.

**Table 1:** Characteristics of the study population.

	Patients who underwent carotid artery ligation	Patients with MMD
Number, n	7	106
Age, mean (SD)	60.9 (11.5)	31.9 (19.6)
< 17 years old, n (%)	0 (0)	33 (31.1)
Female, n (%)	5 (71.4)	72 (67.9)
Follow-up period (months), mean (SD)	50.3 (33.1)	NA
Carotid canal maximum diameter (mm), mean (SD)	5.46 (1.20) *	4.69 (0.69)
Carotid canal perpendicular diameter (mm), mean (SD)	5.04 (0.98) *	4.21 (0.65)
ICA maximum outer diameter (mm), mean (SD)	NA	3.23 (0.65) ^†^
ICA perpendicular outer diameter (mm), mean (SD)	NA	2.99 (0.63) ^†^
Unilateral case, n (%)	NA	23 (21.7)
Suzuki’s stage, n of hemispheres (%)		
Stage 0	NA	18 (8.49)
Stage 1	NA	13 (6.13)
Stage 2	NA	41 (19.3)
Stage 3	NA	64 (30.2)
Stage 4	NA	32 (15.1)
Stage 5	NA	23 (10.8)
Stage 6	NA	21 (9.91)

MMD = moyamoya disease; SD = standard deviation; ICA = internal carotid artery.

* Measured on the surgical side before carotid ligation.

^†^ Measured in 126 hemispheres of 63 patients.

**Fig 1:**
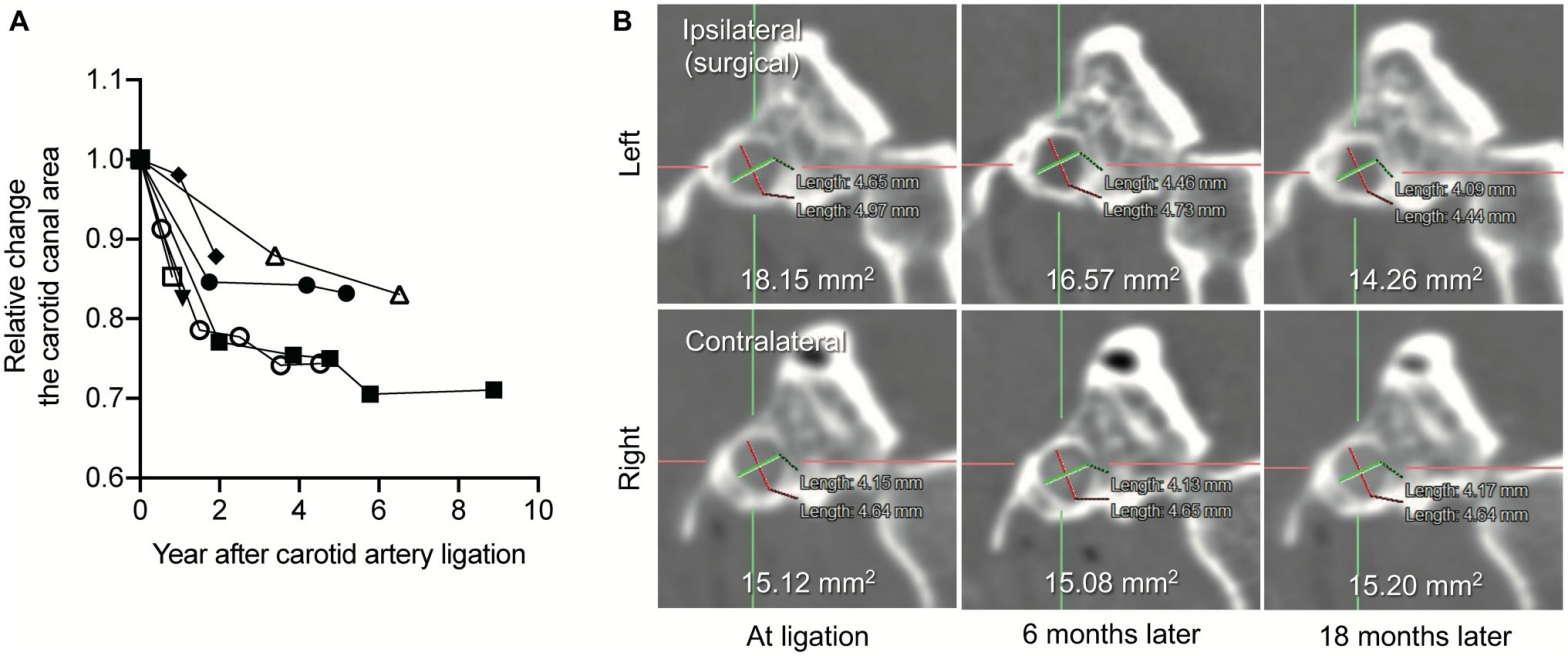
The bony carotid canal narrows after carotid artery ligation. (A) shows the carotid canal area measured in seven patients who underwent carotid artery ligation. The area decreases continuously over years in all cases, with relative changes of 12.2%–28.9%, demonstrating carotid canal plasticity. (B) shows a representative case who underwent carotid artery ligation. Six months after surgery, the carotid canal area on the surgical side had narrowed from 18.15 mm^2^ to 16.57 mm^2^; 18 months after surgery, it had narrowed to 14.26 mm^2^. In contrast, the carotid canal area of the contralateral side did not change.

We then tested the plasticity of the bony carotid canal. We retrospectively reviewed seven patients who underwent carotid artery ligation. The carotid canal area was measured over time after ICA ligation, with a mean follow-up duration of 50.3 months. The canal narrowed in all cases (Fig 1A), showing the plasticity of the bony carotid canal. The narrowing began within 1 year (minimum: 6 months) and continued over several years. The diameter decreased 12.2%–28.9% during the mean follow-up period of 4.2 years. A representative case is shown in Fig 1B. Six months after surgery, the carotid canal area on the surgical side had narrowed from 18.15 mm^2^ to 16.57 mm^2^; 18 months after surgery, it had narrowed to 14.26 mm^2^. In contrast, the carotid canal area of the contralateral side did not change.

### Carotid canal narrowing during contralateral progression of unilateral MMD

**Fig 2:**
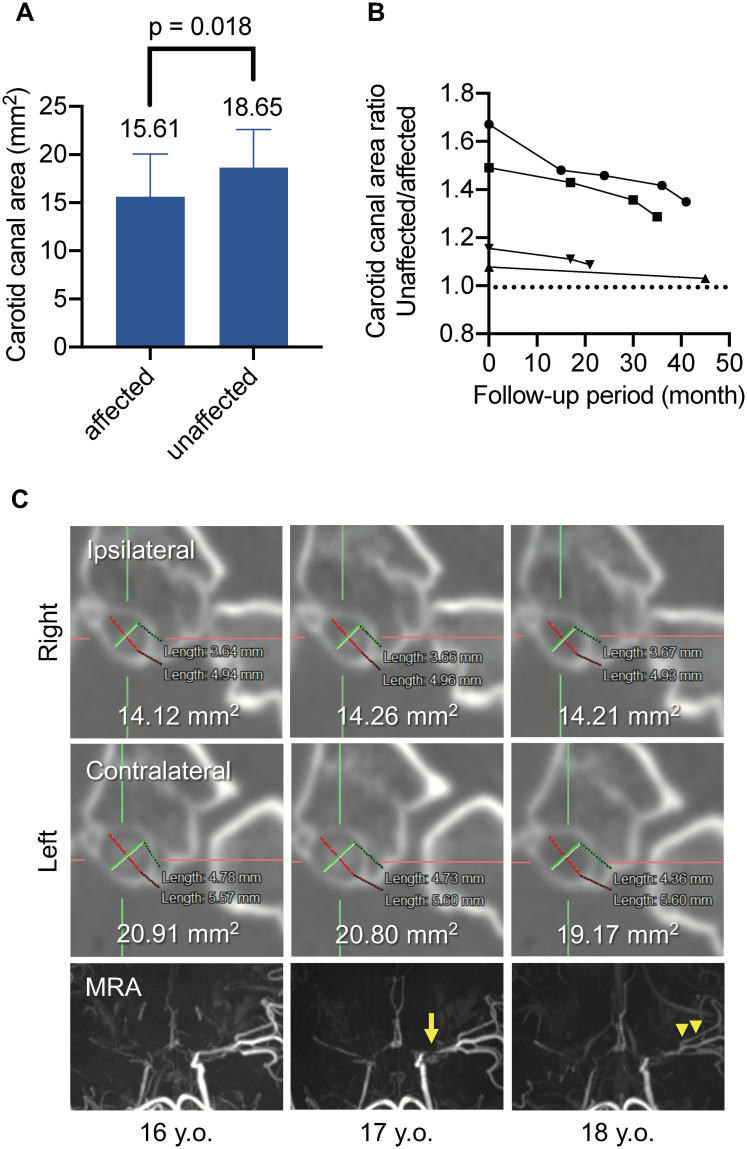
The carotid canal area is larger on the unaffected side than on the affected side in patients with unilateral moyamoya disease, but the difference decreases according to the contralateral progression. (A) shows the carotid canal area compared between the affected side and the unaffected side in patients with unilateral moyamoya disease, and the unaffected side is significantly wider than the affected side (p = 0.018). (B) shows the carotid canal area measured serially in four patients who experienced contralateral progression. The first CT was performed at diagnosis, and the second CT was performed after contralateral progression. Subsequent CT scans were performed when further progression occurred. The carotid canal on the opposite side; i.e., the ipsilateral or affected side, is used as an internal control, and the ratio of unaffected/affected side of the carotid canal area is calculated. The ratio decreases according to the development of contralateral ICA stenosis in all cases. (C) shows a representative case of contralateral progression of unilateral moyamoya disease. Contralateral progression was first detected at the age of 15 years. At 16 years of age, magnetic resonance angiography showed narrowing of the anterior cerebral artery. At the age of 17 years, magnetic resonance angiography showed stenosis of the proximal portion of the middle cerebral artery (arrow). At the age of 18 years, arterial signals in the distal middle cerebral artery decreased (arrowheads). The carotid canal area on the affected side remained unchanged or showed a subtle increase from 14.12 mm^2^ to 14.21 mm^2^, whereas that on the unaffected side decreased from 20.91 mm^2^ to 19.17 mm^2^ according to the contralateral progression of the stenosis around the terminal portion of the ICA.

We also examined longitudinal changes in the bony carotid canal in patients with MMD. We focused on patients with unilateral MMD who had contralateral progression because disease progression can be unambiguously diagnosed and because the opposite side without disease progression can be used as an internal control. In 23 patients with unilateral MMD, the bony carotid canal area on the affected side was significantly narrower than that on the unaffected side (Fig 2A, p = 0.018). Among these unilateral cases, five cases showed contralateral progression, and we analyzed data for four cases who underwent serial CT examinations. The carotid canal area ratio of the unaffected side/affected side was 1.08–1.67 before disease progression, and the ratio approached 1.0 after contralateral progression (Fig 2B). A representative case is shown in Fig 2C. The patient was a 16-year-old man, and the carotid canal on the affected side slightly widened with age. In contrast, the carotid canal on the unaffected side narrowed according to the progression of the ICA stenosis. This case also demonstrated that the carotid canal was negatively remodeled.

### Carotid canal narrowing and negative ICA remodeling

**Fig 3:**
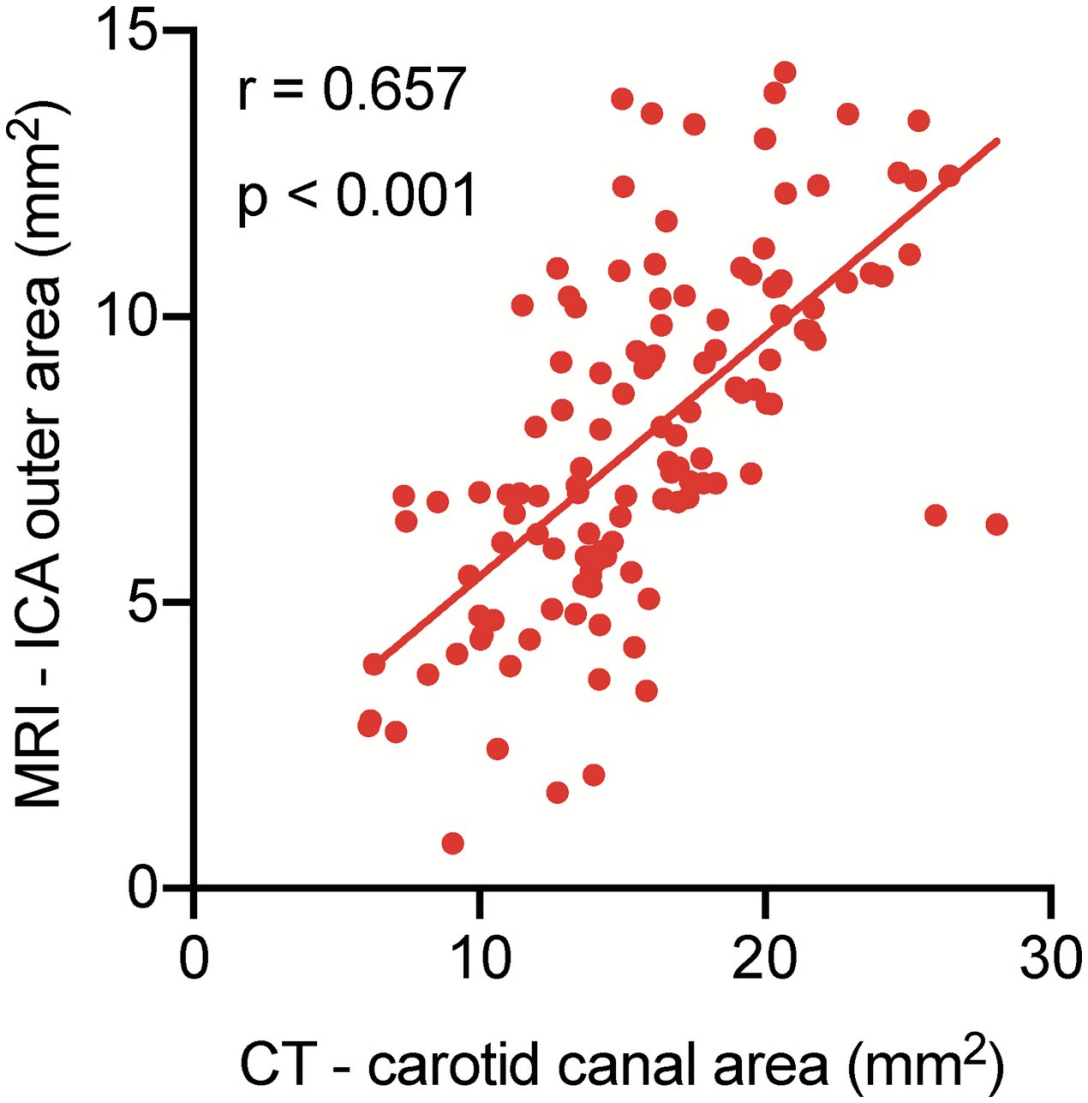
The carotid canal area shows a linear correlation with the outer area of the internal carotid artery. The carotid canal area is assessed by thin-slice CT, and the outer area of the ICA is assessed by high-resolution black-blood MRI in 126 hemispheres of 63 patients with moyamoya disease. There is a significant linear correlation between the carotid canal area and the outer area of the ICA (r = 0.657, p < 0.001).

As MMD is known to be associated with a smaller outer diameter of the ICA, we suspected that carotid canal narrowing was correlated with negative remodeling of the ICA. Next, we examined the correlation between the carotid canal area and the outer area of the ICA measured by high-resolution black-blood MRI in 63 patients with MMD. We confirmed that both variables follow normal distribution (S2 Fig). As expected, the carotid canal area showed a significant linear correlation with the outer area of the ICA (r = 0.657, p < 0.001; Fig 3).

**Fig 4:**
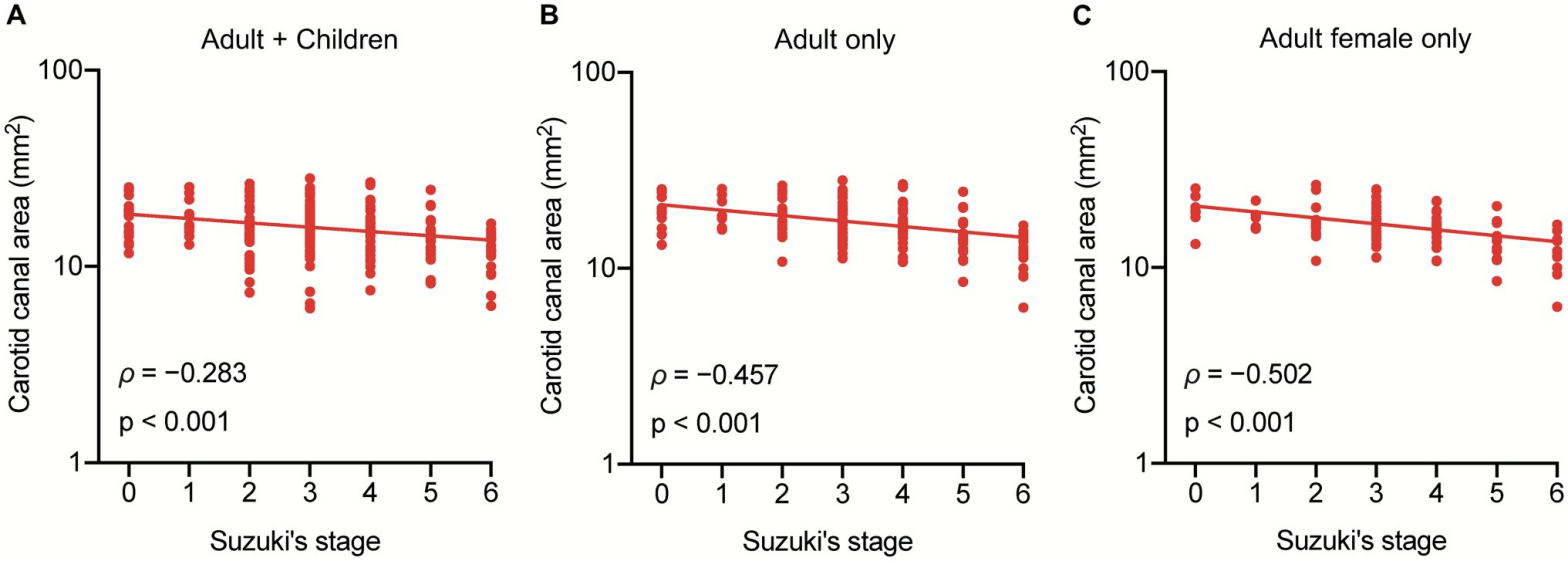
The carotid canal area is negatively correlated with Suzuki’s stage. (A) shows the distribution of the carotid canal area according to Suzuki’s stage in 106 patients with moyamoya disease. The image shows a significant inverse correlation in the carotid canal area with Suzuki’s stage (*ρ* = −0.283, p < 0.001). (B) shows adult-only analysis. There is an apparent inverse correlation with Suzuki’s stage (*ρ* = −0.457, p < 0.001). (C) shows adult female-only analysis. There is also an apparent inverse correlation with Suzuki’s stage (*ρ* = −0.502, p < 0.001).

We further analyzed the association of the carotid canal area with the disease stage. The carotid canal area showed a significant inverse linear correlation with Suzuki’s stage (*ρ* = −0.283, p < 0.001; Fig 4A). Because the previous study has shown that the carotid canal is influenced by age and it is narrower in children [8], we also conducted an adult-only analysis. Adult-only analysis showed an apparent linear correlation with Suzuki’s stage (*ρ* = −0.457, p < 0.001; Fig 4B). We also analyzed the effect of sex on the carotid canal area, showing that female had narrower carotid canal area than male (S3 Fig). Thus, we further conducted an adult female-only analysis, which also showed an apparent linear correlation with Suzuki’s stage (*ρ* = −0.502, p < 0.001; Fig 4C). The maximum diameter and its perpendicular diameter of the carotid canal also showed a significant inverse linear correlation with Suzuki’s stage, respectively (S4 Fig). We also confirmed that the outer area of the ICA was negatively correlated with Suzuki’s stage (*ρ* = −0.402, p < 0.001; S5 Fig). Altogether, our findings suggest that the outer area of the ICA narrows according to disease progression, which is followed by negative remodeling of the bony carotid canal.

## Discussion

We showed that the bony carotid canal narrowed after carotid artery ligation, and that the narrowing began within 6 months after ligation, demonstrating that the bony carotid canal is highly plastic. As carotid artery ligation causes an artificial change in the ICA, we also analyzed the morphological changes in the bony carotid canal in patients with MMD. In patients with unilateral MMD who developed contralateral progression, the carotid canal area on the unaffected side decreased according to the disease progression, compared with the opposite side. This finding supports the hypothesis that the bony carotid canal negatively remodel.

We expected that narrowing of the carotid canal would be caused by negative ICA remodeling. Thus, we analyzed the association of the outer area of the ICA with carotid canal area. As expected, in patients with MMD, the carotid canal area showed a linear correlation with the outer area of the ICA. Both the ICA outer area and the carotid canal area decreased according to the disease progression, suggesting that progression of ICA stenosis induces negative remodeling of the ICA in patients with MMD, followed by, or paralleled by, narrowing of the bony carotid canal.

Importantly, the narrowing of the bony carotid canal in adulthood indicates that the narrow carotid canal does not necessarily mean that the ICA is originally hypoplastic. The plasticity of the bony carotid canal indicates that the canal diameter cannot be used to diagnose hypoplastic ICA. Likewise, even if patients with MMD have a narrow carotid canal, it does not necessarily mean that the ICA was congenitally narrow or that ICA narrowing began in childhood. Watanabe et al. reported that adult patients with MMD had significantly narrower bony carotid canals than control individuals [8]. The authors recognized that the patients had carotid canal hypoplasia, and they speculated that ICA stenosis began in childhood. Other studies also reported the existence of carotid canal hypoplasia in patients with MMD [9–11]. However, our study indicated that a narrowed carotid canal does not necessarily demonstrate a hypoplastic ICA or that growth of the ICA slowed in childhood. Rather, changes in the carotid canal reflect morphological changes in the ICA, and the canal can remodel even in adulthood.

MRI assessment of ICA morphology in patients with MMD was first described by Komiyama et al. [12] using constructive interference in steady-state or heavy T2 MRI, and Kaku et al. [13] then used this method to measure the outer diameter of the ICA. Recent studies used high-resolution MRI (HR-MRI), such as T2 proton-density-weighted imaging, which can assess the morphology of the arterial wall [4, 14, 15]. Longitudinal measurement by HR-MRI will likely be used to assess the outer diameter of the ICA in future studies, but long-term follow-up data are currently unavailable because HR-MRI is a relatively new technology. In this context, the bony carotid canal has advantages regarding longitudinal assessment of ICA morphology. We already had long-term follow-up data for the bony carotid canal, in this study, which made it possible to perform a historical prospective analysis. By using these data, we showed that the bony carotid canal narrows continuously according to disease progression in patients with unilateral MMD, indirectly demonstrating negative remodeling of the ICA. Interestingly, our data showed that negative remodeling of the ICA appears to occur parallel to progression of middle cerebral artery stenosis. However, the concept of negative remodeling of the ICA in patients with MMD should be further confirmed by direct serial measurement of the ICA using HR-MRI.

There are several limitations in this study. First, we did not have longitudinal data regarding the outer diameter of the ICA measured by HR-MRI because this is a relatively new technology. Additionally, the interval of longitudinal CT examinations was long; i.e., almost 1 year. Therefore, we could not determine the exact timing and speed of the negative remodeling of the ICA or the bony carotid canal. Our data showed only that bony carotid canal narrowing could occur within 6 months to a year after carotid artery stenosis or occlusion. A longitudinal study using HR-MRI is needed to assess the patterns of negative remodeling of the ICA and the carotid canal more precisely. Second, the size of the study population, especially the number of patients with unilateral MMD who developed contralateral progression, was small. This is because (1) only 15% of patients with MMD are unilateral cases, and only one-third of patients with unilateral MMD develop contralateral progression [16]; (2) it takes many years for unilateral MMD patients to develop contralateral progression; and (3) not all patients undergo CT imaging repeatedly as CT is not routinely performed multiple times, and because it is unethical to obtain CT images only for research purposes. Most patients with unilateral MMD who did not show contralateral progression underwent only one CT examination. Although the size of the study population was small, the present study provided novel evidence that the carotid canal can change its shape, reflecting the outer area of the ICA.

## Conclusion

Our data showed that the bony carotid canal can be narrower even in adulthood according to the narrowing of the ICA. Therefore, ICA hypoplasia cannot be diagnosed only by a narrow carotid canal. Instead, carotid canal area can be used to longitudinally monitor morphological changes in the ICA.

## Supporting information

S1 Fig The representative images of the diameter measurements of the carotid canal and the ICA. The carotid canal of the horizontal part is sectioned tangentially, and the maximum axial diameter and its perpendicular axial diameter are measured using thin-slice CT and a workstation (AquariusNetStation Terarecon Inc., San Mateo, CA, USA). Using these diameters, the carotid canal area is calculated by applying a modified formula for the area of an ellipse, where area = (maximum diameter) x (perpendicular diameter) x pi/4.
(TIF)

S2 Fig Both the carotid canal area and the outer area of the ICA follow normal distribution. The normality of two continuous variables, the carotid canal area and the outer area of the ICA is checked by the normal Q-Q plot and Shapiro-Wilk test. For both variables, the normal Q-Q plot appears as roughly a straight line, and the p-value is above 0.05.
(TIF)

S3 Fig The carotid canal area is narrower in children or female than in others. (A) The carotid canal area is significantly narrower in children than in adults (mean 12.90 mm^2^ vs 17.16 mm^2^, p < 0.001). It’s also significantly narrower in female than in male (mean 15.16 mm^2^ vs 17.26 mm^2^, p = 0.002). (B) The outer area of the ICA is significantly narrower in children than in adults (mean 6.85 mm^2^ vs 8.47 mm^2^, p = 0.003). The area in female tends to be narrower than in male, but there is no significant difference (mean 7.64 mm^2^ vs 8.34 mm^2^, p = 0.208).
(TIF)

S4 Fig The maximum diameter and its perpendicular diameter of the carotid canal are negatively correlated with Suzuki’s stage. (A) shows the distribution of the carotid canal maximum diameter according to Suzuki’s stage in 106 patients with moyamoya disease. The image shows a significant inverse correlation in the carotid canal area with Suzuki’s stage (*ρ* = −0.249, p < 0.001). (B) shows the distribution of the perpendicular diameter. There is also an apparent inverse correlation with Suzuki’s stage (*ρ* = −0.316, p < 0.001).
(TIF)

S5 Fig The outer area of the ICA is negatively correlated with Suzuki’s stage. (A) shows the distribution of the outer area of the ICA according to Suzuki’s stage in 63 patients with moyamoya disease who had a high-resolution black-blood MRI examination. There is a significant inverse correlation of the outer area of the ICA with Suzuki’s stage (*ρ* = −0.402, p < 0.001). (B) shows adult-only analysis. There is an apparent inverse correlation with Suzuki’s stage (*ρ* = −0.547, p < 0.001). (C) shows adult female-only analysis. There is also an apparent inverse correlation with Suzuki’s stage (*ρ* = −0.505, p < 0.001).
(TIF)

S1 Table Acquisition parameters for CT and HR-BB-MRI. (DOCX)
