# Supplementary figures and images for: Plasticity of the bony carotid canal and its clinical use for assessing negative remodeling of the internal carotid artery
Source: PLoS One. 2021 Dec 15;16(12):e0261235. doi: 10.1371/journal.pone.0261235 (PMC8673643; doi:10.1371/journal.pone.0261235)

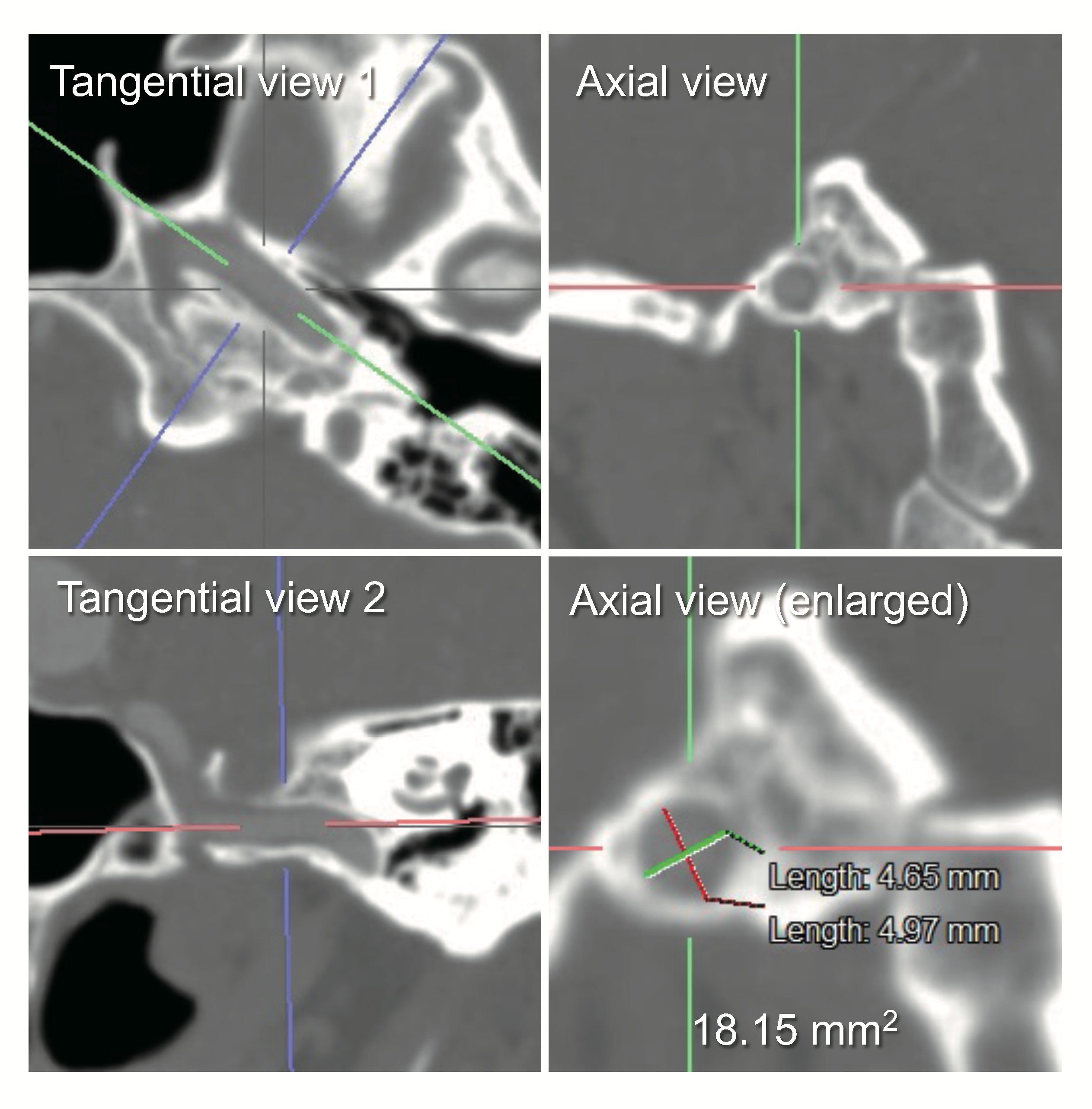

Supplement: S1 Fig — The carotid canal of the horizontal part is sectioned tangentially, and the maximum axial diameter and its perpendicular axial diameter are measured using thin-slice CT and a workstation (AquariusNetStation Terarecon Inc., San Mateo, CA, USA). Using these diameters, the carotid canal area is calculated by applying a modified formula for the area of an ellipse, where area = (maximum diameter) x (perpendicular diameter) x pi/4. (TIF) [file pone.0261235.s001.tif]

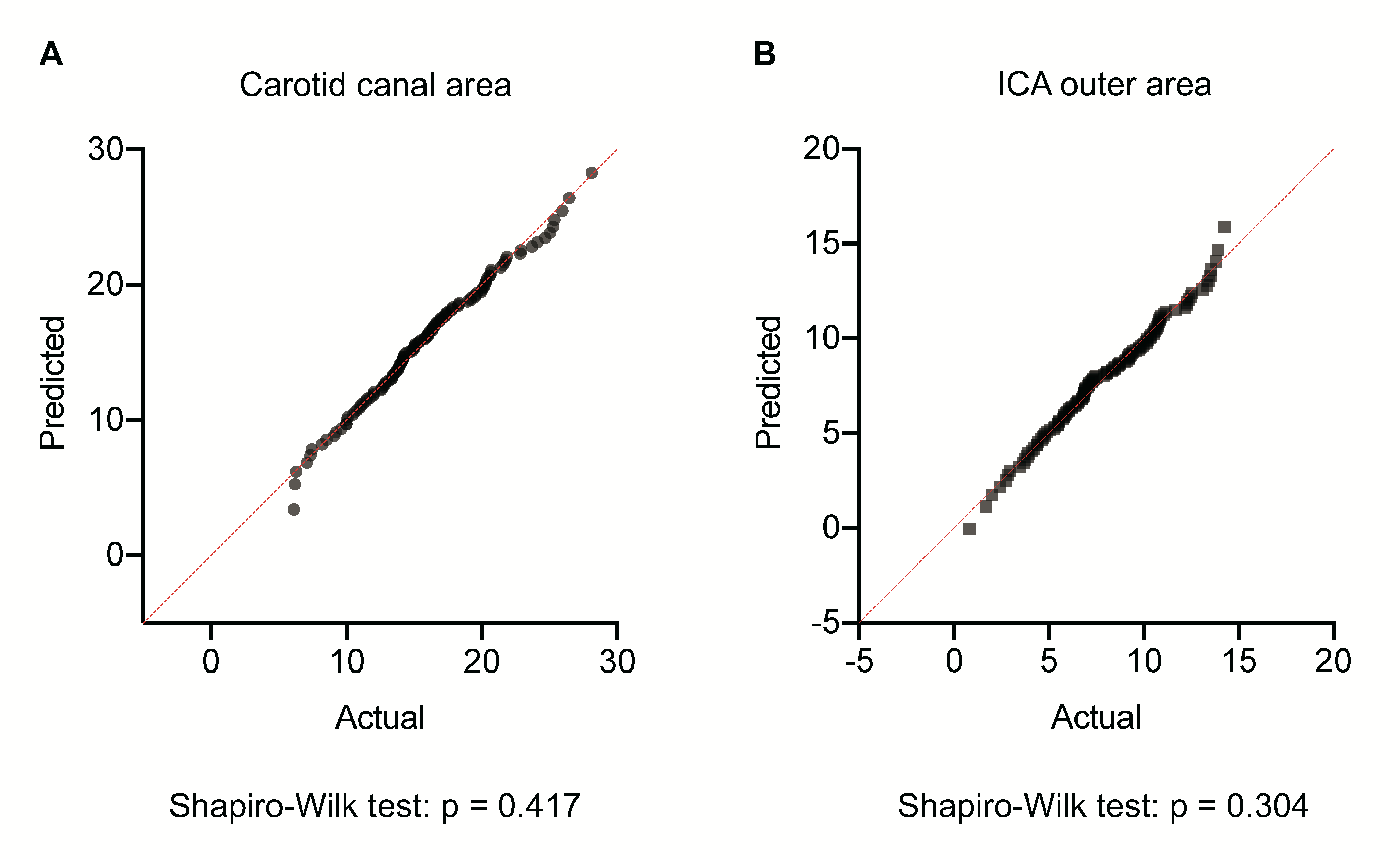

Supplement: S2 Fig — The normality of two continuous variables, the carotid canal area and the outer area of the ICA is checked by the normal Q-Q plot and Shapiro-Wilk test. For both variables, the normal Q-Q plot appears as roughly a straight line, and the p-value is above 0.05. (TIF) [file pone.0261235.s002.tif]

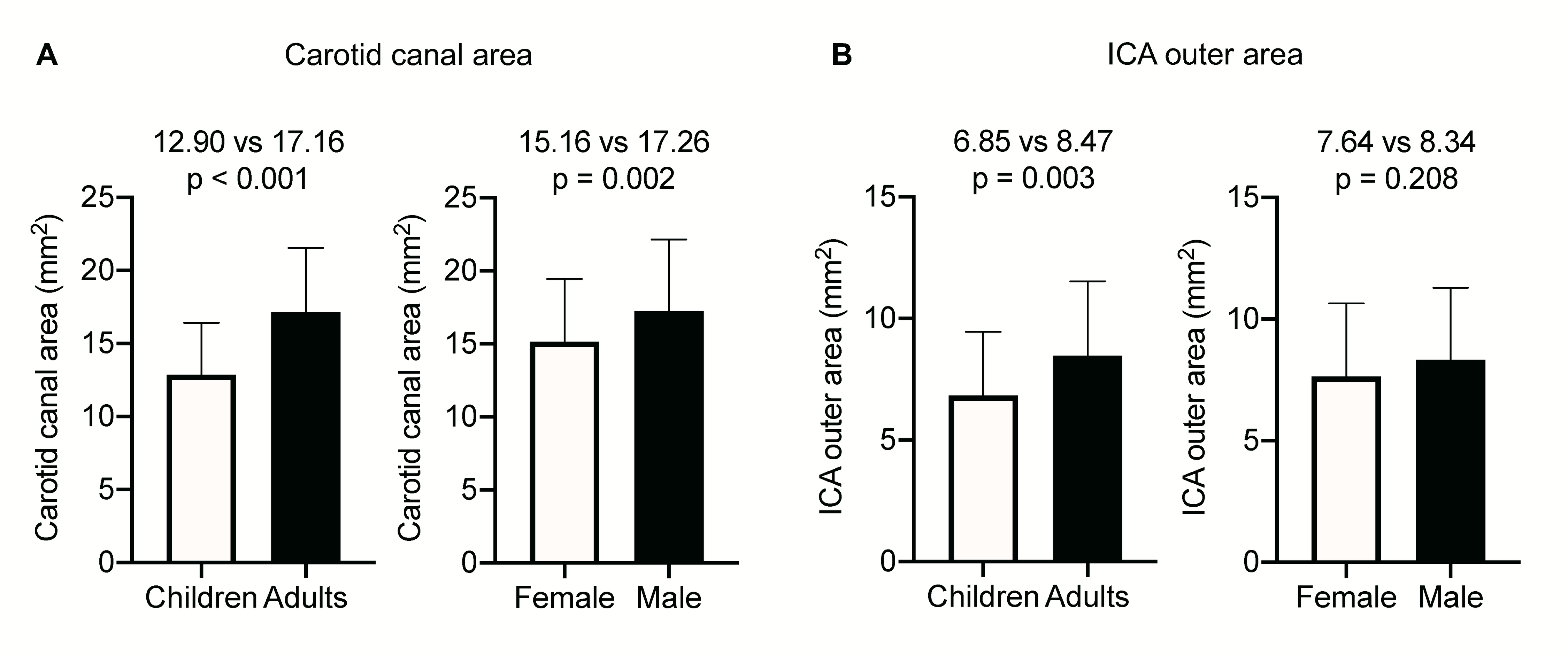

Supplement: S3 Fig — (A) The carotid canal area is significantly narrower in children than in adults (mean 12.90 mm2 vs 17.16 mm2, p < 0.001). It’s also significantly narrower in female than in male (mean 15.16 mm2 vs 17.26 mm2, p = 0.002). (B) The outer area of the ICA is significantly narrower in children than in adults (mean 6.85 mm2 vs 8.47 mm2, p = 0.003). The area in female tends to be narrower than in male, but there is no significant difference (mean 7.64 mm2 vs 8.34 mm2, p = 0.208). (TIF) [file pone.0261235.s003.tif]

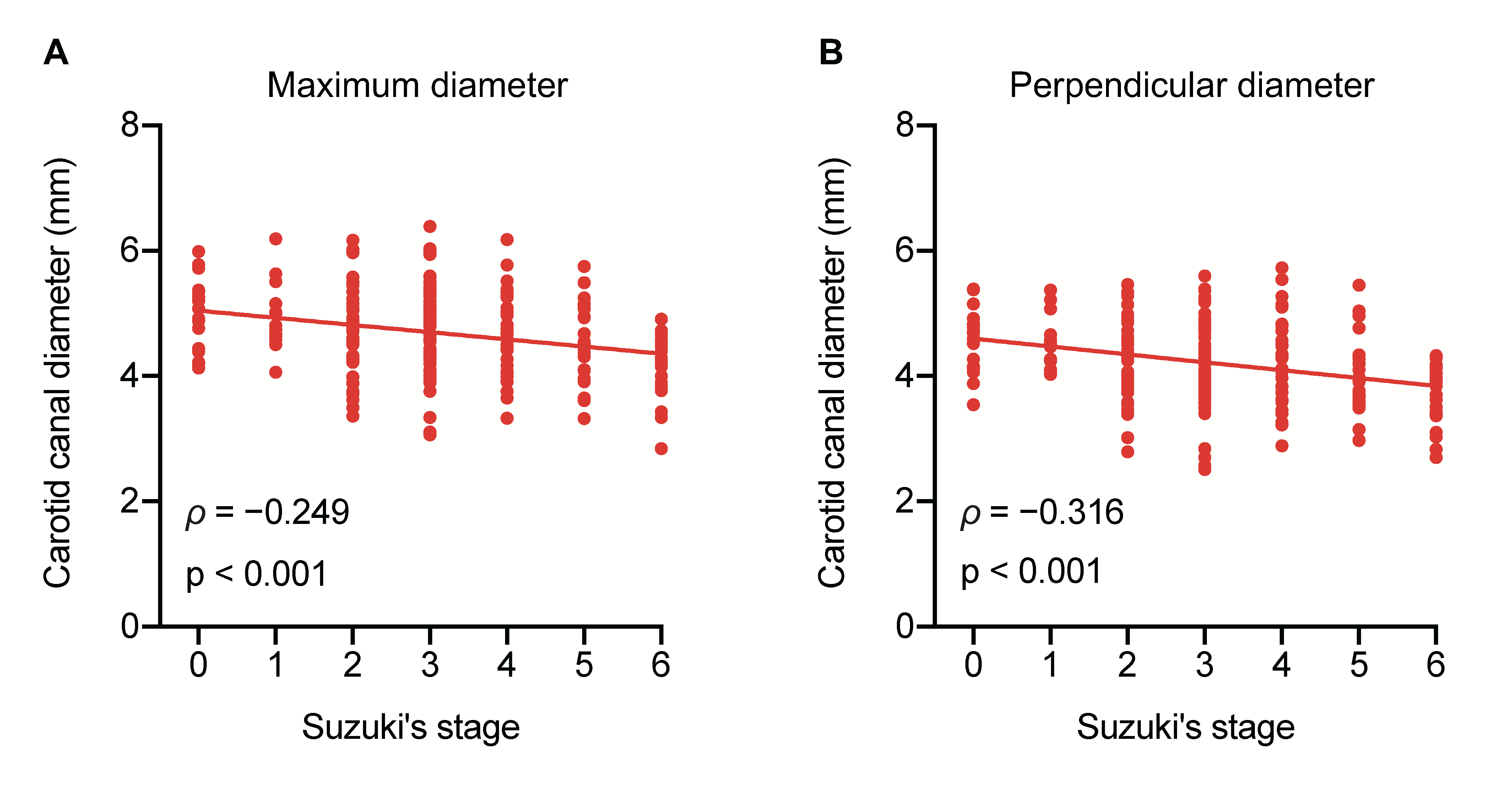

Supplement: S4 Fig — (A) shows the distribution of the carotid canal maximum diameter according to Suzuki’s stage in 106 patients with moyamoya disease. The image shows a significant inverse correlation in the carotid canal area with Suzuki’s stage (ρ = −0.249, p < 0.001). (B) shows the distribution of the perpendicular diameter. There is also an apparent inverse correlation with Suzuki’s stage (ρ = −0.316, p < 0.001). (TIF) [file pone.0261235.s004.tif]

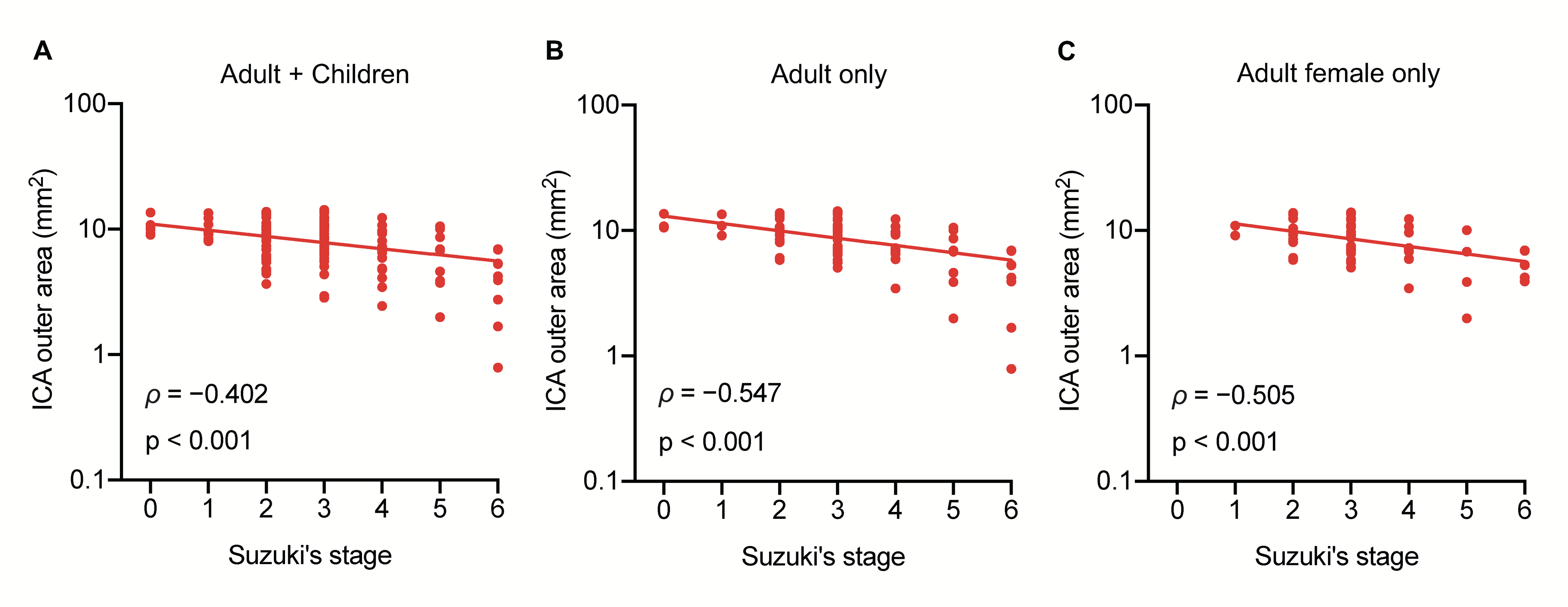

Supplement: S5 Fig — (A) shows the distribution of the outer area of the ICA according to Suzuki’s stage in 63 patients with moyamoya disease who had a high-resolution black-blood MRI examination. There is a significant inverse correlation of the outer area of the ICA with Suzuki’s stage (ρ = −0.402, p < 0.001). (B) shows adult-only analysis. There is an apparent inverse correlation with Suzuki’s stage (ρ = −0.547, p < 0.001). (C) shows adult female-only analysis. There is also an apparent inverse correlation with Suzuki’s stage (ρ = −0.505, p < 0.001). (TIF) [file pone.0261235.s005.tif]
