# Supplementary material for: Plasticity of the bony carotid canal and its clinical use for assessing negative remodeling of the internal carotid artery
Source: PLoS One. 2021 Dec 15;16(12):e0261235. doi: 10.1371/journal.pone.0261235 (PMC8673643; doi:10.1371/journal.pone.0261235)
Supplement: S1 Table — (DOCX) [file pone.0261235.s006.docx]

**S1 Table. Acquisition parameters for CT and HR-BB-MRI.**

| Parameter | CT | HR-BB-MRI |
| --- | --- | --- |
| Pixel size (mm) | 0.41 × 0.41 | 0.56 × 0.56 |
| Slice thickness (mm) | 0.25 | 0.56 |
| Slice gap (mm) | 0 | 0 |
| Matrix | 512 × 512 | 320 × 320 |
| TR (msec) | NA | 1000 |
| TE (msec) | NA | 11 |
| FA | NA | Variable Flip Angle |

CT = computed tomography; HR-BB-MRI = high-resolution black-blood magnetic resonance imaging; TR = repetition time; TE = echo time; FA = flip angle
